# Value of the toluidine blue test as an aid to determine the biopsy site in actinic prurigo cheilitis

**DOI:** 10.4322/acr.2021.247

**Published:** 2021-03-12

**Authors:** Lily Margoth Cedeño-Suárez, María Elisa Vega-Memije, Juan Carlos Cuevas-González, Adalberto Mosqueda-Taylor

**Affiliations:** 1 General Hospital “Dr. Manuel Gea González, Department of Dermatology, Mexico City, Mexico; 2 Autonomous University of Ciudad Juárez, Biomedical Sciences Institute, Stomatology Department, Ciudad Juárez, Chihuahua, México; 3 Autonomous Metropolitan University Xochimilco, Department of Health Care, Mexico City, Mexico

**Keywords:** Actinic Prurigo, toluidine blue O-polyacrylamide polymer, Tertiary Lymphoid Structures

## Abstract

Actinic prurigo (AP) is a type of photodermatosis that primarily affects the Latin American mestizo population. Histologically, AP cheilitis exhibits acanthosis with spongiosis and vacuolation of the basal cell layer overlying a dense lymphocytic inflammatory infiltrate that forms well-defined lymphoid follicles. Toluidine blue is a thiazide, acidophilic, and metachromatic dye used *in vivo* to selectively stain the acidic components of tissues such as sulfates, carboxylates, and phosphate radicals that are incorporated into DNA and RNA. It is necessary to develop a method that allows detecting, on clinical grounds the area of the lesion in which it is more feasible to find such structures. Thus to increase the sensitivity of the biopsy, in AP cheilitis to accurately identify where the lymphoid follicles reside, based on the higher concentration of DNA in such structures and thus confirm the diagnosis. In this study, staining was positive in 85% of patients with AP cheilitis, in 14 of whom 82% lymphoid follicles were observed by histopathology. One of the pathologist’s problems in establishing the diagnosis of AP is that the main histopathological characteristics are not always identified in the submitted samples because it is not easy to clinically identify the most representative site of the lesion selected for performing a biopsy. Based on our results, we propose using toluidine blue as an auxiliary method to choose a tissue sample to facilitate the diagnosis and allow clinicians to make clinical correlations between the histopathological and therapeutic findings.

Actinic prurigo (AP) is a type of photodermatosis that primarily affects the Latin American mestizo population. AP is related to genetic susceptibility, specifically in the HLA-DR_4_ allele, which varies between populations.^1^

The skin lesions in AP are polymorphic and include erythematous macules, papules that converge and form plaques, serohematic crusts, lichenification areas, scarring and hypopigmentation or residual hyperpigmentation. Further, lower lip involvement at the vermillion border is common in a high percentage of the affected individuals and maybe the only expression of the disease, when is commonly termed as AP cheilitis; in this location, it is characterized by edema, cracks, fissures, ulcers that are covered by serohematic crusts, erythema and areas of residual hyperpigmentation. that is associated with intense pruritus that extends to the adjacent skin.^1^

Histologically, AP cheilitis exhibits acanthosis with spongiosis and vacuolation of the basal cell layer overlying a dense lymphocytic inflammatory infiltrate that forms well-defined lymphoid follicles (comprising B lymphocytes in the center and T lymphocytes at the periphery), macrophages, eosinophils, and mast cells (Figure 1).^2^

**Figure 1:**
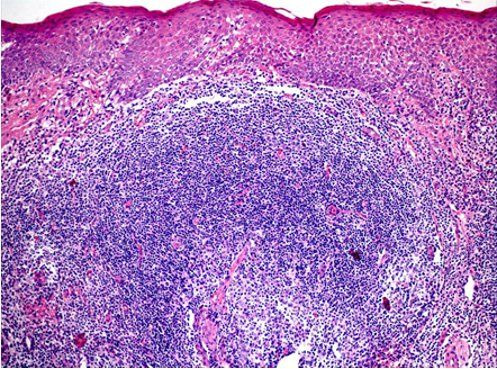
Histopathological features of actinic prurigo cheilitis.

AP cheilitis affects 85% of patients and is the sole manifestation of the disease in 27% of those who are affected.^2^ Diagnosis of AP when there is only lip involvement requires an assessment of its clinical and histopathological characteristics, and very often, it is necessary to take several tissue samples to demonstrate the presence of lymphoid follicles, which are considered an essential element to confirm the nature of this disorder.

Toluidine blue is a thiazide, acidophilic, and metachromatic dye used *in vivo*, to selectively stain the acidic components of tissues such as sulfates, carboxylates, and phosphate radicals that are incorporated into DNA and RNA.^3^^,^^4^ Therefore, as epithelial dysplasia and carcinomas contain higher amounts of DNA and RNA than adjacent normal epithelium. Toluidine blue has been used as an ancillary method for identifying areas with increased cell proliferation in lesions for which there is a clinical suspicion of malignancy.^5^

As only 63.8% of biopsies of AP cheilitis have lymphoid follicles,^2^ it is necessary to develop a method that allows detecting, on clinical grounds, the lesion area. It is more feasible to find such structures, to increase the sensitivity of the biopsy. Consequently, we sought to determine the value of toluidine blue as an ancillary method in obtaining tissue samples in AP cheilitis to accurately identify where the lymphoid follicles reside, based on the higher DNA concentration in such structures and thus confirm the diagnosis.

We included 20 patients (11 women and 9 men) aged between 7 and 45 years (mean 25.7) who presented clinically with AP cheilitis (Figure 2A). The detection technique comprised the application of toluidine blue with a swab along the affected vermillion border. After 60 seconds, the site was cleaned with 2% acetic acid solution and rinsed with water or saline solution, leaving clearly defined an area in which the dye persisted visible in the tissue (Figure 2B).

**Figure 2:**
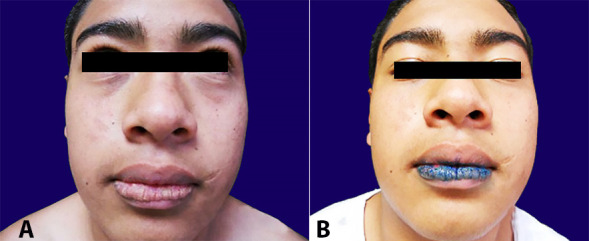
**A –** Clinical aspects of actinic prurigo cheilitis; **B –** Persistent staining with toluidine blue after removing dye with 2% acetic acid (positive zone).

This region was then injected with 0.5 cc of 2% xylocaine solution with epinephrine, distal to the area where the incisional biopsy was performed, which included a spindle-shaped fragment of tissue of approximately 5 mm in length, fixed in 10% formaldehyde, and processed for hematoxylin and eosin staining.

## DISCUSSION

Toluidine blue is already used to identify malignant/premalignant lesions in the oral mucosa; although no risk of malignant transformation has been reported in actinic prurigo cheilitis, it can be considered as an aid to decide the site to take the biopsy due to the dense lymphocytic inflammatory infiltrate that forms well-defined lymphoid follicles pick up the dye.^6^ In this study, staining was positive in 17/20 (85%) patients with AP cheilitis, in 14 of whom (82%) lymphoid follicles were observed by histopathology. In the 3 cases in which there were no lymphoid follicles, the inflammatory infiltrate was mild to moderate, while in the remaining 3 cases that were negative for toluidine blue, no inflammatory cells were seen. These findings contrast with what has been reported in previous studies in which lymphoid follicles were noted in only 63.8% of samples,^2^ submitted to biopsy without application of toluidine blue, indicating the greater sensitivity of this technique to confirm the diagnosis.

One of the pathologist’s problems to establish the diagnosis of PA is that the main histopathological characteristics are not always identified in the submitted samples because it is not easy to clinically identify the most representative site of the lesion selected for performing a biopsy.

## CONCLUSION

Based on our results, we propose using toluidine blue as an auxiliary method to choose a tissue sample to facilitate the diagnosis and allow clinicians to make clinical correlations between the histopathological and therapeutic findings.
